# Systematic Studies on the Antioxidant Capacity and Volatile Compound Profile of Yellow Mealworm Larvae (*T. molitor* L.) under Different Drying Regimes

**DOI:** 10.3390/insects13020166

**Published:** 2022-02-03

**Authors:** Claudia Keil, Sandra Grebenteuch, Nina Kröncke, Fenja Kulow, Sebastian Pfeif, Clemens Kanzler, Sascha Rohn, Georg Boeck, Rainer Benning, Hajo Haase

**Affiliations:** 1 Department of Food Chemistry and Toxicology, Institute of Food Technology and Food Chemistry, Technische Universität Berlin, Straße des 17. Juni 135, 10623 Berlin, Germany; fenja.kulow@yahoo.de; 2 Department of Food Chemistry and Analysis, Institute of Food Technology and Food Chemistry, Technische Universität Berlin, Straße des 17. Juni 135, 10623 Berlin, Germany; sandra.grebenteuch@tu-berlin.de (S.G.); pfeifsebastian@gmail.com (S.P.); clemens.kanzler@tu-berlin.de (C.K.); rohn@tu-berlin.de (S.R.); 3 Institute for Food and Environmental Research e. V., Papendorfer Weg 3, 14806 Bad Belzig, Germany; 4 Institute of Food Technology and Bioprocess Engineering, University of Applied Sciences Bremerhaven, An der Karlstadt 8, 27568 Bremerhaven, Germany; nkroencke@hs-bremerhaven.de (N.K.); rbenning@hs-bremerhaven.de (R.B.); 5 GloMic GmbH, Krampnitzer Weg 102, 14089 Berlin, Germany; g.boeck@glomic.de

**Keywords:** *Tenebrio molitor* L., drying, nutrient composition, fatty acids, volatile compounds, antioxidant capacity, lipid oxidation, Maillard reaction, flavour deterioration

## Abstract

**Simple Summary:**

Global population growth will increasingly challenge the food industry sector in the coming years. The yellow mealworm is a promising candidate for production on an industrial scale and it is also the first insect to be approved by EFSA in 2021 under the EU`s Novel Food Regulation. Drying is an important preservation step in the industrial processing of insects but there is limited information on the stability of nutritional, bioactive and sensory components during drying. Thus, this study sought to investigate the impact of different drying procedures (freeze-drying, microwave drying, infrared drying, oven drying and high frequency drying) on the chemical composition, antioxidant capacity and volatile profile of mealworm larvae. To summarize: (1) Mealworm larvae contain considerable amounts of polar or nonpolar extractable antioxidants, whose molecular identity needs to be to be clarified in the future. (2) The drying process was decisive with regard to the extraction efficiency of these antioxidants. The highest antioxidant capacities were found in high-temperature dried larvae, while the extracts obtained from the freeze-dried larvae had the lowest values. (3) The analyses of volatile compounds provide information on the extent to which the Maillard reaction and lipid oxidation occur and indicate relevant changes in the chemical composition during the drying procedure. Deepening the knowledge of process-induced changes of mealworm quality will contribute to improve *Tenebrio molitor* L. processing technologies, a basic prerequisite for utilizing mealworms as novel food or animal feed in the future.

**Abstract:**

The yellow mealworm (*Tenebrio molitor* L., Coleoptera: Tenebrionidae) is an edible insect and due to its ubiquitous occurrence and the frequency of consumption, a promising candidate for the cultivation and production on an industrial scale. Moreover, it is the first insect to be approved by EFSA 2021 following the Novel Food Regulation. Industrial production of mealworms necessitates optimized processing techniques, where drying as the first postharvest procedure is of utmost importance for the quality of the final product. The focus of the present study was to analyse the chemical composition, antioxidant capacity, volatile compound profile and colouring of mealworm larvae dried in various regimes (freeze-drying, microwave drying, infrared drying, rack-oven drying and high-frequency drying). Proximate composition and fatty acid profile were similar for all dried larvae. Freeze dried larvae were predominantly marked by lipid oxidation with significantly higher peroxide values, secondary/tertiary oxidation products in the headspace GC-MS profiles and lower antioxidant capacity. High-temperature treatment in the rack oven—and to some extent also infrared or microwave drying—led to mealworm larvae darkening and the appearance of volatile Maillard secondary products such as 2-methylpropanoic acid, 2-/3-methylbutanoic acid and alkylpyrazines. High-frequency drying as a new emerging technology in insect processing was the most cost-effective method with energy costs of solely 0.09 Є/kg *T. molitor* L. leading to final larval material characterized by both lipid oxidation and nonenzymatic Maillard-browning.

## 1. Introduction

The demand for food needed to feed the world’s population in 2100 far exceeds current production capacities. United Nations projections expect nearly 11 billion people on Earth in 2100—about 3 billion more— to feed than 2021 [1]. Current food production systems will not be able to sustainably supply the projected world population with conventional animal protein, especially in the face of climate change [2,3,4]. Animal proteins require more natural resources to be produced, so future food capacities at medium levels of animal-based agriculture are much more desirable from a nutritional as well as sustainability perspective [5,6]. Insects have received attention as a sustainable, macro- and micronutrient-rich food and feed resource over the past 10 years [7,8,9]. The 2013 FAO report on global insect consumption [10] was ground-breaking, as it gave credibility to the acceptance of edible insects in the western industrial society. Even though insect products have not yet become mainstream in the western world, recent trends show a global increase in the numbers of insect manufacturing companies and consumers. In terms of volume, the value of the global edible insect market will pass 8 billion USD by 2030, with a total volume of 730,000 tons of insect-based products (whole insects, insect ingredients and products incorporated with edible insects) on the global market to be expected [11,12]. Commercial challenges facing today’s insect industry include reducing production costs, facilitating certifications and regulations, improving marketing, expanding retail and distribution and expanding processing into consumer-friendly products. The international marketing of insect products is yet restricted due to inconsistent regulations for the approval of insects or insect products worldwide. While the USA, Canada, New Zealand and Australia have comparatively lenient national legislation, the EU has classified edible insects as “novel foods”, necessitating a tedious authorization procedure for production and distribution [13,14]. In 2021, three kinds of insects were positively evaluated by the European Food Safety Authority (EFSA) and received legal status for EU marketing—amongst them dried yellow mealworm larvae *(Tenebrio molitor* L., Coleoptera: Tenebrionidae) [15,16]. Along with the black soldier fly (*Hermetia illucens*), the lesser mealworm (*Alphitobius diaperinus*), the house fly larvae (*Musca domestica* L.) and various grasshopper species, mealworm larvae are the most promising insect food and feed replacement alternatives that are currently being studied [12,14,17,18]. *T. molitor* breeding and harvesting ranges nowadays from small to large-scale automated mass approaches [18,19,20] and almost any of the steps from insect rearing to the edible products is addressed from economical, food safety and nutritional quality perspectives [15,16,21,22]. Mealworms are generally high in protein (30–65% of dry matter), carbohydrates (2.2–11.5% of dry matter), fat (10–30% of dry matter) and particularly rich in unsaturated fatty acids [9,23]. This macro-composition and the high moisture content of fresh larvae with a water activity near to 1 favours lipid oxidation and non-enzymatic Maillard browning reactions already during the drying procedure [24]. The Maillard pathway, a complex chemical cascade that starts from a chemical reaction between amino components and reducing sugars, produces numerous and mostly highly reactive ketones, aldehydes, dicarbonyl and heterocyclic compounds, as well as high-molecular-weight melanoidins [25,26,27]. They all contribute to the colour, flavour, nutritional and antioxidant properties of heat-treated foods. Likewise, lipid oxidation also generates numerous primary, secondary and tertiary oxidation products that influence shelf life and product properties such as taste, texture, mouthfeel and appearance [28,29]. In complex matrices-such as mealworm larvae-intermediates of the Maillard reaction and lipid oxidation will likely pass into interrelated routes and thus contribute in a coordinated manner to the properties of the dried insect [30]. During industrial processing, mealworm larvae are first killed by heat or cold treatment and then dried to slow down enzymatic and non-enzymatic degradation and microbiological spoilage. The energy and time requirements, as well as the intended post-processing of the insects, are important criteria when deciding on a particular drying technique. Still drying must be carried out properly to reduce potential microbial, chemical and allergenic hazards, while ensuring nutritional quality and consumers preference in terms of texture, taste and flavour [31]. Oven drying and freeze drying are currently most common for insects on an industrial scale [32]. Freeze-drying is also implemented in the updated IPIFF (International Platform of Insects for Food and Feed) Guide on Good Hygiene Practices for European Union producers of insects as food and feed [33]. EFSA confirmed the safety for frozen, freeze-dried as well as thermally dried mealworms [15,16]. In addition to these common industrial drying procedures, other innovative, seminal drying methods [32,34] have been investigated on *T. molitor* larvae for their effects on macro/micronutrient composition and accessibility, colour changes, protein solubility and lipid oxidation prevalence [24,35,36,37,38,39,40]. Drying devices based on microwave-, infrared- or fluid bed drying are already used industrially [32,41]. The scope of our study was to compare some of these established drying methods (freeze drying, oven drying, microwave drying, infrared drying) and high frequency drying as an emerging technology in insect processing, with regard to their effects on larvae nutritional value (water activity, nutrient composition), lipid oxidation, total antioxidant capacity, volatile component profile and mealworm larvae colouring. These results will help to further advance the processing technologies for *T. molitor* larvae, which is of immense importance considering the future prospects of insects for food and feed.

## 2. Materials and Methods

### 2.1. Insect Samples

*T. molitor* larvae of own breeding were cultured at the University of Applied Sciences Bremerhaven, as described previously [24]. Mealworms were reared for a period of 16–18 weeks in a rearing room at 25 °C with a relative humidity of 55–60% and fed with wheat bran *ad libitum* after hatching. At harvest time, larvae were separated from wheat bran and frass and frozen at −21 °C for 48 h (HAS 47520, Beko Grundig Deutschland GmbH, Neu-Isenburg, Germany) before drying. The larvae rearing and the accomplishment of the experiments were carried out under consideration of the German Animal Welfare Act.

### 2.2. Drying Procedures

Five different methodologies were applied for *T. molitor* larvae drying: freeze-drying, microwave drying, infrared drying, rack oven drying or high frequency drying. For freeze-drying, a total of 500 g frozen larvae material was subdivided onto five freezing plates and placed within a freeze-dryer (Christ Beta 1–8, Martin Christ Gefriertrocknungsanlagen GmbH, Osterode am Harz, Germany). The condenser was set to −50 °C prior to drying and a vacuum was applied for 27 h, with final temperatures of around 20 °C at the shelves. Microwave drying was carried out in a conventional microwave (H 6100, Miele & Cie. KG, Gütersloh, Germany). Frozen larvae (150 g) were placed as a thin layer on the plate (45 × 35 × 3 cm) in the middle of the microwave and dried for 15 min. Infrared drying of 1 kg mealworm larvae material was carried out in the laboratory-built device IRD-A 70/90-22-Batch-K (perfect solutions GmbH, Meiningen, Germany) at 130 °C for 15 min under evaporation. For rack oven drying 400 g of frozen larvae were subdivided onto two baking plates (60 × 80 × 2 cm) in the middle of the rotating convection oven (RI 1.0608-TL, MIWE Michael Wenz GmbH, Germany) and dried at 60 °C, ventilation stage 2 for 19 h. A 13.56 MHz pilot-scale GloMic-Laborofen GR1000 radio frequency dryer (GloMic GmbH, Berlin, Germany) equipped with multiple controllable heat sources (high-frequency heating, P 2 kW; conventional electric heating, P 1kW; electric infrared heating P 1kW) was used for high-frequency drying in this study. 540 g of frozen larvae placed in a trough were heated within 230 s (load power 300 Watt) to 120 °C inside the drying chamber. Subsequently, the larvae were dried in a non-equidistant program under temperature maintenance until the weight loss was below 0.2g/Wh (see Supplementary Figure S1 for further details of HF-drying). After drying, the larvae were separately packed in closed polyethylene bags and stored until analysis at 5 °C in a climatic chamber (HPP 110, GmbH + Co. KG, Schwabach, Germany). 

Energy consumption (EC) of the different drying methods were calculated based on the assumption that the equipment run at maximal capacity during drying according to Saifullah et al. 2019 [42]. EC for infrared drying and rack oven drying was calculated using Equation (1), where D_Temp_ is the drying temperature used (°C), M_Temp_ is the maximum temperature (°C) and MO the maximum energy output (kW) for the drying equipment and t is the drying time (h).

(1) $$EC=\frac{D_{Temp}}{M_{Temp}}\times MO\times t$$

The EC calculation for freeze-drying and microwave drying (Equation (2)) is based on the supplied electrical power (P in kW) and the time required for drying (t in h).

(2) EC=P×t

In the high-frequency application, the EC is based on the continuous measurement of the forward and reflected power by high-frequency power detectors. The difference of these two quantities is the time-dependent absorbed power. The EC is the time integral over the absorbed power. In addition, the reflected power is permanently minimized by an automatic RF matchbox.

### 2.3. Proximate Analysis

Proximate composition of fresh or dried *T. molitor* larvae was determined as described before [24]. The moisture content was analysed after heating ground insect material in a drying oven (U10, GmbH + Co. KG, Schwabach, Germany) for 4 h at 103 °C. Water activity (a_w_-value) was measured using a water activity meter (LabMaster aw neo; Novasina AG, Lachen, Switzerland) until the water activity was stable for 15 min. Kjeldahl procedure was used for protein quantification applying a nitrogen-to-protein conversion factor of 6.25 according to DIN EN 25663 and the Association of German Agricultural Analytic and Research Institutes. Total fat content was determined from ground larvae material according to the Soxhlet method (VDLUFA, 1976). The ash content was analysed as described by VDLUFA, 1976 [43].

### 2.4. Colour Evaluation

Colour parameters were determined from whole-body larvae digital images according to a protocol from de Oliveira et al. [44] using Photoshop CS4 Version 11.0. After background correction, the larvae bunch was scaled to 800*800 pixels and five different areas per bunch marked by the eyedropper tool to 101*101 pixels were analysed for CIELAB parameters. These coordinates are the lightness of the colour (L* = 0 yields black and L* = 100 indicates diffuse white), the values of components a* (green-red axis) and b* (blue-yellow axis) are between −128 and +128, respectively. Based on these values, total colour differences (ΔE^∗^) between mealworm samples were calculated with $\Delta E=\sqrt{a^{*2}+b^{*2}+L^{*2}}$ [38].

### 2.5. Fatty Acid Composition and Fat Oxidation

Lipids were extracted from fresh and dried ground mealworm larvae by methanol/chloroform (1:2 *v/v*)) extraction [45] and subsequently subjected to trimethylsulfonium hydroxide derivatization. The fatty acid composition was measured as described in Kröncke et al. (2019) [37] using a gas chromatography-flame ionization detection device (GC-2025, Shimadzu Deutschland GmbH, Duisburg Germany) on a DB-23 column (60 m × 0.25 mm × 0.25 µm; Agilent Technologies Deutschland GmbH, Munich, Germany). Fatty acid methyl esters were identified by comparison of their retention times with fatty acid methyl ester standards (Sigma Aldrich Chemie GmbH, Taufkirchen, Germany). The peroxide value (POV) was determined according to the Wheeler method [46] in a microanalytical approach. 0.1 g of the oily phase was weighed into an Erlenmeyer flask and mixed with 30 mL of solvent (acetic acid/chloroform 3:2) and 0.5 mL of saturated potassium iodide solution. After stirring for 60 s, 30 mL distilled water was added and titrated potentiometrically (TitroLine^®^ 7000, Pt 61 electrode, Xylem Analytics Germany Sales GmbH & Co. KG, Weilheim, Germany with sodium thiosulphate (0.001 N).

### 2.6. Volatile Compound Measurements

Three different headspace-based sample extraction techniques (static headspace (static HS), headspace solid phase microextraction (HS-SPME) and headspace in-tube extraction (HS-ITEX)) [47], combined with gas chromatography-mass spectrometry (GC–MS) were used to cover the broadest possible spectrum of volatile compounds from mealworm larvae. Approximately 1 g of whole-body fresh or dried larvae, without further pre-processing, was used for each of the extractions. All methods were initially optimized to ensure the lowest possible thermal stress during headspace extraction and sufficient sensitivity. All extraction procedures were automatically executed using Combi-PAL-RSI autosampler (Axel Semrau GmbH & Co. KG, Sprockhövel, Germany). In case of static HS, the agitator module was incubated for 7 min at 120 °C to carry volatile compounds directly into the vapor phase. Subsequently, 1 mL of vapor space was injected into the GC−MS system. In HS-SPME mode samples were incubated in the agitator for 6 min at 100 °C. A PDMS/CAR/DVB fibre was exposed to the headspace of the sample to allow volatile compound enrichment for 15 min and then desorbed for 1 min in the injector (230 °C). For the dynamic HS-ITEX (Tenax TA trapping adsorbent), the optimized parameters are an agitator temperature of 100 °C, an incubation period of 1 min, 30 stroke cycles, extraction volume 1000 µL/stroke and 60 μL/s extraction speed. The trap was desorbed at 200 °C with a desorption speed of 50 µL/s. All gas-chromatography analyses were conducted using a GC−MS system consisting of a GC-17A gas chromatograph (Shimadzu Deutschland GmbH, Duisburg, Germany) and a Shimadzu GCMS-QP5000 mass detector. The volatile compounds were separated using a Restek GmbH (Bad Homburg, Germany) Rtx^®^-Volatiles column (60 m × 0.25 mm, 1 μm film thickness). The following settings were used: carrier gas, helium; flow 1.00 mL/min; splitless injection; injection temperature 230 °C; interface temperature 230 °C; ion source temperature 200 °C; ionization energy 70 eV; temperature gradient 40 °C for 5 min, 10 °C/min to 150 °C, 2 °C/min to 198 °C. Chemical identification was carried out by comparing retention times and mass spectra of samples with those of commercial standards (Sigma-Aldrich, Munich, Germany) and data available from NIST library (National Institute of Standards and Technology, Gaithersburg, MD, USA). The quantitation was performed in SCAN mode (mass scan *m*/*z* 33–350) using total ion current (TIC) and expressed as abundance units (AU) and provide semiquantitative data. Data acquisition was performed using the GCMS solution software version 1.20 (Shimadzu Deutschland GmbH, Duisburg, Germany).

### 2.7. Antioxidant Analysis

A total of 0.5 g of the fresh or dried mealworm larvae were mixed with 5 mL of solvents of different polarity (H_2_O, PBS, ethanol, methanol, acetonitrile, MeOH/CHCl_3_ 2:1 (*v/v*), MeOH/CHCl_3_ 3:1 (*v/v*)), dispersed (30 sec, 13,500 min^−1^) using T25 digital ULTRA-TURRAX^®^ (IKA^®^-Werke GmbH & CO. KG, Staufen, Germany) and extracted by agitation (neoLab^®^ Rotator 2-1175, neoLab Migge GmbH, Heidelberg, Germany) under light-protection for 30 min at room temperature. Subsequently, the samples were centrifuged in the Eppendorf^®^ Centrifuge 5810 (Eppendorf, Hamburg, Germany) at 2500 g for 10 min. Part of the supernatants was either freeze-dried (Alpha 2–4 freeze-dryer, Martin Christ Gefriertrocknungsanlagen GmbH, Osterode, Germany) or applied to nitrogen blow down evaporation (MULTIVAP nitrogen blow down evaporator; Dräger Medical Deutschland GmbH, Lübeck, Germany) to determine the extraction efficiency. Depending on their polarity, the extracts were used in different antioxidant assays to determine their total antioxidant capacity [48]. 

The ABTS [2,2′-azino-nis (3-ethylbenzthiazoline-6-sulfuric acid)] radical cation scavenging capacity (TEAC Assay) was determined as described by Kanzler et al. (2017) [49]. The ABTS^•+^ was prepared by mixing an aqueous 10 mM ABTS solution and a 3.5 mM potassium peroxodisulfate solution in a 1:1 ratio. Before use, the mixture was incubated overnight at room temperature in the dark and diluted in PBS buffer (4.9 mM phosphate, 150mM NaCl, pH 7.2) to an absorbance of A_734_ ~1. 100 µL ABTS^•+^ solution and the same volume of the 1:100-prediluted extracts or Trolox calibration standards (0.010−0.100 mM in either PBS buffer or ethanol) were combined in 96-well plate cavities. The ABTS^+•^ bleaching was monitored at 734 nm and the decolouration after 30 min was used as the measure of antioxidant capacity. Intrinsic absorbance of the extracts was also recorded by measuring the absorption when adding the solvent instead of the ABTS^•+^ radical solution. According to Apak et al. (2016) [50] radical scavenging capacities were expressed as µmol of Trolox equivalents (TE) per gram of mealworm. 

1,1-diphenyl-2-pircydrazyl (DPPH) radical scavenging assays [51] were carried out in an experimentally similar way by mixing equivalent volumes of 400 µM DPPH^•^ (made in methanol, A_519_ ~1) and 1:250-prediluted lipophilic larvae extracts and determination of the absorbance at 519 nm after a 30 min incubation period. α-tocopherol (0.010−0.100 mM; dissolved from 2.5 mM stock in the respective extraction agent) was used as lipophilic reference antioxidant to calculate DPPH radical scavenging capacity expressed as µmol α-tocopherol equivalents (αTocE) per gram of mealworm. 

The Folin–Ciocalteu antioxidant assay (FCR assay) was performed according to a protocol published by Ainsworth and Gillespie (2007) [52]. 50 µL of 20% (*v/v*) Folin–Ciocalteu agent was premixed with either 50 µL of 10-fold diluted mealworm extracts or gallic acid standards (0.010−0.100 mM; dissolved from 1mM stock in the respective extraction agent) followed by the addition of 150 µL 850 mM NaCO_3_. The A_765_ values measured after 30 min incubation were used for calculating the antioxidant capacity expressed as gallus acid equivalents (GAE) per gram of mealworm.

### 2.8. Statistical Analysis

Statistical significance of the experimental results was calculated by GraphPad prism software (version 8.0, GraphPad Software, San Diego, CA, USA). All experiments were conducted in at least three independent replicates as indicated in the figure legends. Data are shown as means ± standard error of mean (SEM). Data were compared between treatments using one-way ANOVA with Tukey’s multiple comparison test, applying a 95% confidence level.

## 3. Results

### 3.1. Energy Consumption and Efficiency of the Drying Methods

Time and energy consumption are critical factors for selection of suitable drying methods as they are linked to the cost of drying. The time requirements in this study followed the order of freeze-drying > rack oven drying > high-frequency drying > microwave drying = infrared drying (see Table 1). The energy and cost calculation of the different drying methods values high frequency drying as the most cost-effective method (energy cost 0.09 EUR /kg larvae material); freeze-drying in comparison consumed about 55 times more energy (energy cost 4.96 EUR /kg larvae material). 

The moisture content measured for undried larvae was around 59.5% with a water activity of 0.69 (see Table 2). In any case, drying resulted in lower moisture levels; the rack oven dried samples had the highest values of 7.87 ± 0.03%. Still, all dried samples reached a_w_ below 0.4, which is sufficient for long term storage. Despite minor variations, protein, fat and fibre values were highly similar for undried and dried samples (see Table 2) and within the ranges found in the literature. 

### 3.2. Colour Analysis

The body colour parameters of dried mealworms are shown in Table 3. Freeze-dried samples showed the highest L* values (lightness) with a red/green coordinate (a* value) of around 12.1 and a blue/yellow colour component (b* value) of around 44.5. Microwave and infrared dried samples shifted significantly toward lower brightness (*p* < 0.001 FD vs. MW; *p* < 0.001 FD vs. IR), increased red values (*p* < 0.001 FD vs. MD; *p* < 0.001 FD vs. ID) and yellowness (*p* < 0.001 FD vs. MD; *p* < 0.001 FD vs. ID). Rack oven and high-frequency dried larvae were profoundly darker (lowest L* values) with less red and yellow components in their colour. Based on the calculated total colour differences the high-frequency dried larvae were most different from freeze-dried (ΔE* value 41.8), next to microwave dried (ΔE* value 39.8), infrared dried (ΔE* value 25.4) and closest to rack oven dried larvae (ΔE* value 8.4)

### 3.3. Fatty Acid Analysis

Overall, medium and long chain fatty acids from 12 to 24 C-atoms were found in the dried larvae (see Table 4). Palmitic acid (16:0) was the major saturated fatty acids (SFA) followed by stearic acid (18:0) and myristic acid (14:0). Regarding the unsaturated fatty acids (UFA), dried mealworm larvae had a high amount of mono-UFA oleic acid (18:1 **ω**-9) and poly-UFA linoleic acid (18:2 ω-6). In addition, lauric acid, arachidic acid, behenic acid, lignoceric acid (SFA), as well as linolenic acid (18:3 ω-3 PUFA) were found, but their percentages were rather low. Other PUFAs, such as eicosapentaenoic acid (EPA) and docosahexaenoic acid (DHA), were not detected in the *T. molitor* larvae of this study. Although the samples differed in the percentage of some fatty acids, they showed an overall favourable PUFA/SFA balance (PUFA/SFA ratios between 0.78 to 2.3) substantiating the nutritional value of their lipids (see Table 4). In addition to fatty acid composition, the peroxide values were determined (see Table 4) to monitor oxidative spoilage, where high POV values may reflect either increased formation of lipid hydroperoxides or its reduced decomposition. Peroxide values of undried larvae were 2.02 ± 0.14 mmol O_2_/kg fat. For high-frequency, rack oven, infrared and microwave dried larvae, values in the range of 0.6 to 2.0 were measured. The freeze-dried samples are far above this (7.22 ± 0.78 mmol O_2_/kg fat), indicating increased lipid oxidation during the freeze-drying process. 

### 3.4. Volatile Compounds

**Figure 1:**
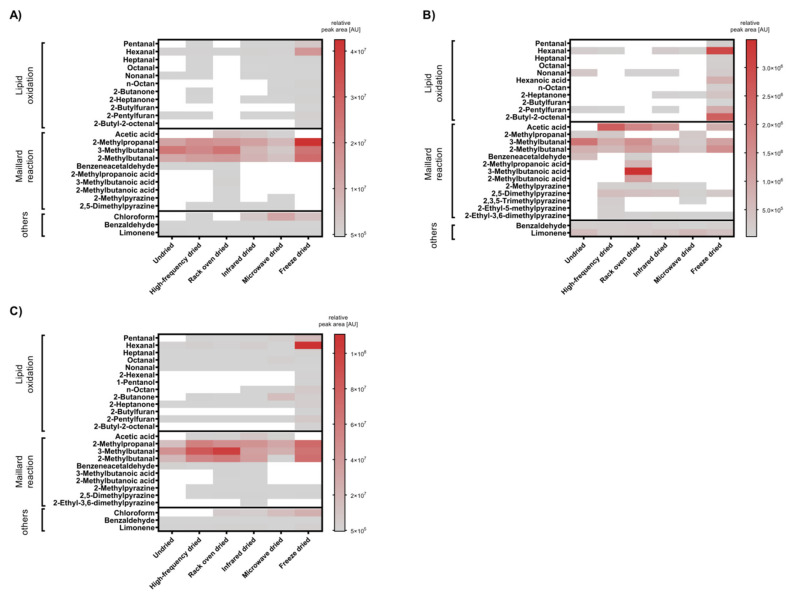
Heatmaps of volatile compounds from mealworm larvae detected by (**A**) static HS (**B**) HS-SPME or (**C**) HS-ITEX GC-MS. Shown are the mean values of the peak area of volatile organic compounds relative to larval weight. (N ≥ 3); white areas= volatile component was not detectable.

A total of 30 volatile compounds were identified from mealworm larvae samples using three different headspace GC-MS based techniques. These included 12 aldehydes, 2 ketones, 5 acids, 1 alcohol, 5 pyrazines, 2 furans, 2 alkanes and 1 terpene. Most of these are compounds occurring in above-average amounts in heat-treated foods and are formed during the Maillard reaction or as secondary and tertiary lipid oxidation products (Figure 1 and Supplementary Tables S1–S3). The Strecker degradation products of the branched-chain amino acids valin (2-methylpropanal), leucin (3-methylbutanal) and isoleucin (2-methylbutanal), although slightly variant depending on the HS-GC methods, were already present in high amounts in the undried larvae. These aldehyde values remained high in the freeze-dried samples. Rack oven-, dielectric- (high-frequency, microwave) and infrared-dried larvae showed comparatively lower mean peak area values for these substances. The headspace GC-MS profiles of the larvae rack oven-dried at 60 °C are rich in Strecker degradation products such as 2-methylpropanoic acid, 2-/3-methylbutanoic acid and alkylpyrazines, indicating the progress in Maillard reactions during this drying procedure. Alkylprazines were also detected in the dielectric heated larvae samples in addition to lipid oxidation markers. The heat maps of freeze-dried samples are dominated by volatile compounds formed in the course of lipid oxidation.

### 3.5. Total Antioxidant Capacity of Mealworm Larvae Extracts

**Figure 2:**
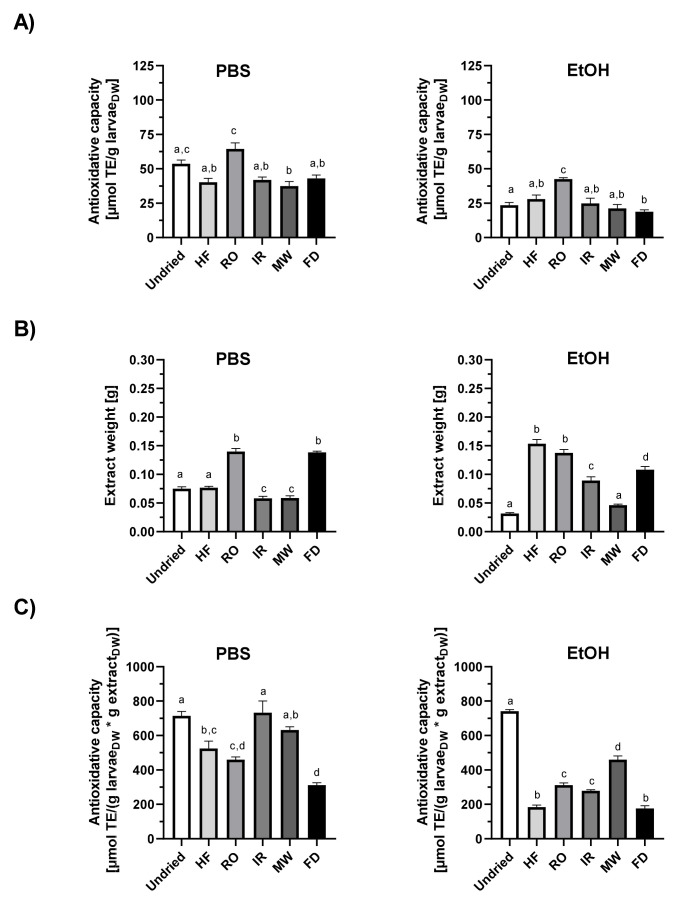
TEAC antioxidant capacity of *T. molitor* larvae extracts. Antioxidant capacity of PBS or ethanol mealworm extracts measured by ABTS^•+^ reduction and normalized to (**A**) the larval dry weight or (**C**) the larval dry weight per extract dry weight. (**B**) Dry weight of extracts obtained from 0.5 g larval material each as a measure of extraction efficiency. Data are shown as means ± SEM of three replicates. Significantly different means within one panel do not share the same letters (analysed by one-way ANOVA with Tukey’s multiple comparison test, *p* < 0.05). DW = dry weight, TE = Trolox equivalents. HF = high-frequency dried, RO = rack oven-dried, IR = infrared dried, MW= microwave dried, FD = freeze-dried.

**Figure 3:**
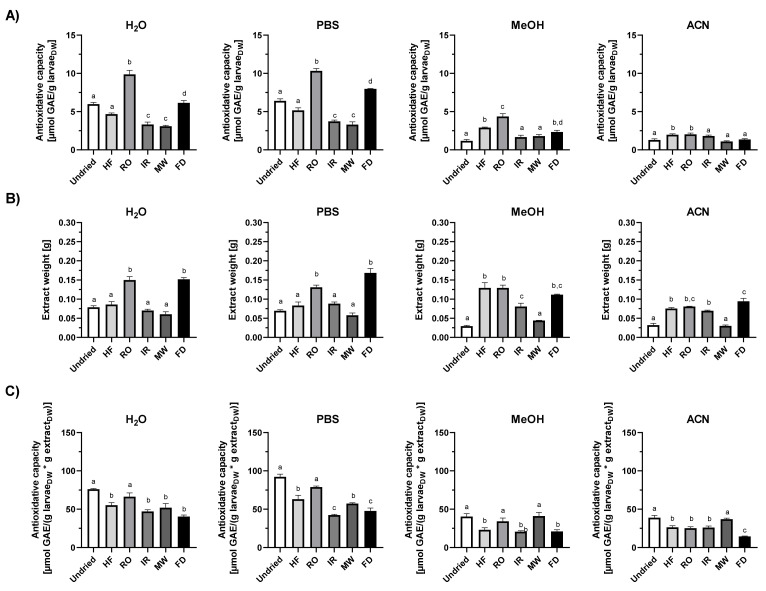
Folin–Ciocalteu reducing capacity of *T. molitor* larvae extracts. Antioxidant capacity of H_2_O, PBS, methanol and acetonitrile mealworm extracts measured by reduction of Folin–Ciocalteu reagent and normalized to (**A**) the larval dry weight or (**C**) the larval dry weight per extract dry weight. (**B**) Dry weight of extracts obtained from 0.5 g larval material each as a measure of extraction efficiency. Data are shown as means ± SEM of three replicates. Significantly different means within one panel do not share the same letters (analysed by one-way ANOVA with Tukey’s multiple comparison test, *p* < 0.05). DW= dry weight, GAE = Gallic acid equivalent. DW= dry weight, TE = Trolox equivalents. HF = high-frequency dried, RO = rack oven-dried, IR = infrared dried, MW = microwave dried, FD = freeze-dried.

**Figure 4:**
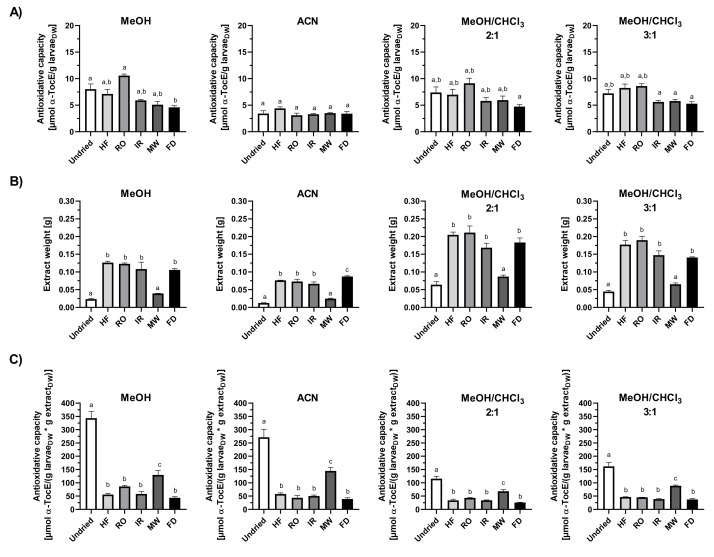
DPPH radical scavenging capacity of *T. molitor* larvae extracts. Antioxidant capacity of methanol, acetonitrile and methanol/chloroform mealworm extracts measured by DPPH^•^ scavenging assay and normalized to (**A**) the larval dry weight or (**C**) the larval dry weight per extract dry weight. (**B**) Dry weight of extracts obtained from 0.5 g larval material each as a measure of extraction efficiency. Data are shown as means ± SEM of three replicates. Significantly different means within one panel do not share the same letters (analysed by one-way ANOVA with Tukey’s multiple comparison test, *p* < 0.05). DW = dry weight, α-TocE= α-tocopherol equivalent. DW = dry weight, GAE = Gallic acid equivalent. DW = dry weight, TE = Trolox equivalents. HF = high-frequency dried, RO = rack oven-dried, IR = infrared dried, MW = microwave dried, FD = freeze-dried.

To get an insight into the activity of lipophilic and hydrophilic antioxidant *T. molitor* larvae were extracted with either polar or nonpolar solvents and the total antioxidant capacities of the extracts were evaluated by TEAC, FCR and DPPH assays, respectively (Figure 2, Figure 3 and Figure 4).

The various antioxidant tests performed confirm both hydrophilic and lipophilic antioxidants in fresh mealworm larvae extracts, in accordance with results of previous studies. However, the extracts were found to have different levels of antioxidative capacity in the test solvent used. Normalized to the larvae dry weight input, PBS extracts from undried larvae exhibited a higher ABTS^●^ scavenging capacity (53.7 ± 4.6 µmol TE/g larvae_DW_) than the ethanol extracts (23.5 ± 1.7 µmol TE/g larvae_DW_) (Figure 2A). Folin–Ciocalteu reducing capacity by dry weight was the highest for the water (6.0 ± 0.3 µmol GAE/g larvae_DW_) and PBS crude larvae extracts (6.4 ± 0.4); values of the methanol and acetonitrile extracts were about 80% lower (Figure 3A). The DPPH^•^ scavenging activity of methanol or methanol/chloroform fractions from undried larvae varied between 4.5 and 5.1 α-TocE/g larvae_DW_, acetonitrile extracts were 3.4 ± 0.8 α-TocE/g larvae_Dw_ (Figure 4A). 

The drying method of the larvae proved to be relevant for the extraction efficiency outcome (Figure 2B, Figure 3, Figure 4B), which is of importance in terms of quantity and diversity of antioxidants in the polar and nonpolar extracts. Thus, for the aqueous solvents (H_2_O and PBS) extraction did work best for rack oven dried and freeze-dried larvae (Figure 2B and Figure 3B). When calculating the antioxidative capacity relative to the larvae material input, RO aqueous extracts scored the highest in TEAC and FCR (Figure 2A and Figure 3A). However, if the factor extract dry weight was included into the calculation of the antioxidant capacity, the values of the fresh as well as the HF-, IR- and MW-dried larvae aqueous extracts adjusted to those of the RO larvae (Figure 2C and Figure 3C). This indicates higher antioxidant quantities and/or potency in the crude, HF-, IR- and MW-dried larvae extracts compared to the RO samples. The freeze-dried larvae were the most favourable matrix in extraction yields for almost all of the solvents used. However, those extracts performed the worst in their relative antioxidant capacities in all tests (Figure 2C, Figure 3, Figure 4C).

## 4. Discussion

Feeding a world population of 11 billion people by 2100 in the face of climate change requires the establishment of food production systems that are sustainable, efficient, nutritious and healthy. More than 2000 insect species are documented as edible worldwide [54]; *T. molitor* is one of the most promising for large-scale industrial production [15,16,18,19] and commercial applications [55,56]. Drying as part of the industrial processing chain is crucial to ensure high quality insect raw material, ingredients or derived products for food and feed [31,32]. The results of the present study confirmed the efficiency of freeze-drying, oven drying, microwave drying and, infrared drying and high-frequency drying in reducing the mealworm larvae moisture content (residual moisture content between 1.38–7.87%) and water activity (final a_w_ 0.11–0.3), which is sufficient for long term storage [57]. Similar drying efficiencies were already reported for mealworm larvae in our recent studies [24,37] and also by others [15,16,38,44,58]. High-frequency heating has been studied to control insect pests in agricultural products for many years [59]; during insect processing it is not yet established. However, the recent research report of the EU-funded SUStainable INsect CHAIN project (SUSINCHAIN) highlights the potential and usability of this emerging technology for insect larvae processing [60]. The drying data for *T. molitor* L. also recommend this method.

The proximate composition for any dried mealworm larvae in this study is comparable to other raw or processed insects [7,8,24,37,38,39,61]. The same applies to the fatty acid pattern, which is similar to what has already been reported for raw or dried *T. molitor* larvae [7,8,38,62]. Their high content of linolenic acid and beneficial PUFA/SFA ratio is favorable with regard to current dietary recommendations for fats [63]. Yet, the FD larvae were the highest in peroxide levels, indicating pronounced lipid oxidation during freeze- drying. The lipid oxidation tendency of mealworm larvae during freeze-drying was also noted in the studies published by Lenarts et al. (POV evaluation) [38] and Kröncke et al. (4-HNE quantification) [24]. The EFSA assessment of thermally dried mealworm larvae quotes POVs between 0.7–16.3 meq O_2_/kg fat for different *T. molitor* L. batches [15]. Freeze-dried larvae had POVs between 1.0–3.2 meq O_2_/kg fat [16]. However, it is unsubstantiated to compare these data with regard to the underlying drying technology, as these data were submitted by two different applicants to EFSA and therefore differences due to *T. molitor* strain specificities, feeding or rearing cannot be excluded. Another indication of the oxidative milieu during freeze-drying is given by this study`s headspace volatile compound screen. The GC-MS profiles of the freeze-dried mealworm larvae were far more enriched in aldehydes (secondary lipid degradation products) as well as 2-alkylfurans and methyl ketones (tertiary lipid degradation products) [29,64]. The Strecker aldehydes 2-methylpropanal, 3-methylbutanal and 2-methylbutanal were already detected in raw larvae, similar to the results of other studies [37,65]. These very likely emanate the enzymatic degradation of branched chain amino acids leucin/isoleucin/valin (Ehrlich pathway) during mealworm larvae rearing [66,67]. The increase in 2-methylpropanal, 3-methylbutanal and 2-methylbutanal in freeze dried larvae is consistent with the role of lipid oxidation-derived carbonyls in the Strecker-like degradation of amino acids and the importance of such amine-carbonyl reactions for food flavors [30,68]. The volatile screen from rack oven dried, high-frequency dried larvae, and to some extent also IR and microwave dried larvae, covers Maillard intermediates; shifting Strecker aldehydes toward subsequent Maillard products, such as 2-methylpropanoic acid, 2-/3-methylbutanoic acid and alkylpyrazines [25,69,70]. It is quite likely that the high temperatures in the rack oven or the high frequency drying system accelerated non-enzymatic Maillard browning. At least this is reflected in the colour evaluation of this study, which shows that the rack oven- and high-frequency dried larvae were the darkest samples. In addition to free amino acids, amino sugars (e.g., d-glucosamine (chitosan monomer) or *N*-Acetyl-d-glucosamine (chitin monomer)) [23,56] or proteins typically found in the larvae/insects [71] might contribute to non-enzymatic browning reactions during drying. The aforementioned study from Grossmann et al. [71] reveals alterations of partially proteolyzed mealworm extracts under Maillard conditions toward more complexity, both in gas-chromatography-olfactometry and in sensory evaluation. Raw mealworms reared on wheat bran until late instar were described as having strong, wet, earthy and less intense, oily, shrimp and sweet corn-like attributes [72,73]. Convection oven treatment (180°C, 5min) led to roasted shrimp-like odour characteristics attributed to intermediates of both Maillard reaction and lipid oxidation, such as pyrazines, ketones, alcohols and aldehydes [73]. Likewise, Zołnierczyk et al. (2021) [74] reported increased pyrazine levels in larvae baked between 160–200 °C. In sensory analysis, these larvae were evaluated as having burnt, toasted bacon and bread-like flavours. These observations highlight how industrial processes involved in the production of mealworm products may affect the volatile quality of the final product. In view of the development of consumer-oriented products, especially in the western world, where insects and insect-based products are increasing as novel foods, it would be desirable to integrate volatile analytics and sensory evaluation into the qualitative assessment of samples from the drying stage onwards. 

In addition to volatile compounds, the Maillard and lipid oxidation cascades generate a variety of redox-active molecules contributing to the pro/antioxidants balance in complex food/feed matrices such as processed insect larvae. The various antioxidant tests performed in the present study confirmed the presence of both hydrophilic and lipophilic antioxidants in wheat bran reared mealworm larvae, as already shown by others [75]. The drying method proved to be decisive for the yield of polar and nonpolar extractables. This led to considerable differences when calculating the antioxidant capacities solely referred to the mealworm dry weight or when including the extract weight in addition. The freeze-dried larvae were the most favourable matrix in terms of extraction yield, but these extracts were significantly lower in antioxidant content and/or potency, which is another indication of the pro-oxidant milieu of freeze-drying. Heat-dried larvae were in-between fresh and freeze-dried larvae in their double-normalized antioxidant values, suggesting that endogenous antioxidants are degraded during drying but also new ones are generated. It is conceivable that high temperatures during drying accelerate non-enzymatic browning, whereby the formation of antioxidative Maillard products inhibits lipid oxidation [30]. This would be a plausible explanation for the increased lipid oxidation tendency of the freeze-dried larvae, which needs to be proven in future experiments. Extraction efficiency has so far been barely considered in antioxidant-related insect processing studies. Most often, the antioxidant capacity is given by the weight of the insect sample, ignoring the actual extraction yield. However, when it comes to evaluating insect larvae of different rearing [76], processing status [77,78] or when comparing them with other food matrices [79], knowledge and consideration of extraction efficiency is indispensable. In fact, the recovery of antioxidants or bioactive molecules from insects—including mealworm larvae—requires the use of specific extraction conditions in terms of the type of solvent and extraction method [56,80]. In the future, it would be worthwhile to investigate the effects of drying processes on the structure of *T. molitor* L., e.g., by means of scanning electron microscopy or X-ray tomography with synchrotron radiation [61], always with the ulterior motive of improving the extraction yield of bioactive substances. Furthermore, it would be of great interest to specify the enzymatic and non-enzymatic antioxidants, especially for those insects intended for food and feed. This study’s antioxidant capacity values determined by TEAC (25–75 µmol TE/g_larvae DW_), DPPH (5–10 µmol αTocE/g_larvae DW_) and FCR assay (4–12 µmol GAE/g_larvae DW_) exceeds the mealworm larvae classical antioxidants Vitamin E/total tocopherol (1.9–55 nmol/g) [76,81] or ascorbic acid content (170–2000 nmol/g) [7,81] by orders of magnitude. Due to their simplicity, TEAC, DPPH and FCR are often used to determine the overall antioxidant status of biological samples. However, in multi-throughput, these methods are not suitable for quality assessment of individual antioxidants in complex food/feed samples [50]. The variety of molecules reactive towards ABTS^●^, DPPH^●^ or the FCR ranges from polyphenols, thiols (GSH), vitamin derivatives (l-ascorbic acid, NADH, retinoic acid, tocopherols/trolox), proteins and amino acids (tyrosine, tryptophan, cysteine), nucleotide bases (guanine), aldehydes from lipid oxidation to reductones, heterocycles and melanoidins from the Maillard reaction cascade [26,82,83,84]. Instrumental setups such as online couplings of liquid chromatography and post-column derivatization with either ABTS^●+^ or DPPH^●^ (online HPLC-UV/Vis TEAC or DPPH methods) combined with HPLC-ESI-ToF-MS [85,86] offer the possibility to unravel the molecular identity of potent mealworm larvae antioxidants and furthermore to assess changes in their specific antioxidant activity as a result of drying, which is of utmost importance from both insect processing as well as nutritive perspective. Recent studies showed the benefits of implementing food industry by-products into mealworm larvae rearing in terms of improved larval growth, diminished microbial load, antioxidants status and ω−3 fatty acid enrichment. The question, which technologies are most suitable to protect polyunsaturated fatty acid-enriched larvae for storage and in subsequent processing steps has not yet been answered. Protection against oxidation of the ω−3 fatty acids could possibly be achieved by enriching the mealworm larvae with antioxidants. Knowing the molecular identity of the mealworm’s endogenous antioxidants and increasing them by means of biofortification would be of immense advantage. Such fortified mealworm larvae can be classified as extraordinarily valuable but require correct processing for the benefit of the consumers.

## 5. Conclusions

The larvae of the yellow mealworm (*Tenebrio molitor* L., Coleoptera: Tenebrionidae), the first insect to be approved by EFSA as a novel food in 2021, is being promoted as a promising environmentally sustainable food source with a significantly lower carbon footprint compared to other animal sources. Summarizing this study’s results, it can be concluded that any of the drying technologies (freeze-drying, oven drying, microwave drying, infrared drying or high frequency drying) were efficient in reducing the larvae moisture without affecting them in proximate composition and fatty acid composition. Freeze-drying made mealworm larvae susceptible toward lipid oxidation, which is reflected by increased peroxide values, diminished antioxidative capacity and a volatility profile enriched in lipid oxidation products. Rack oven-, dielectric- (high-frequency dried, microwave dried) and infrared-dried larvae were much more Maillard-imprinted in the headspace GC-MS volatile screens and darker in body colouring. In terms of energy and time consumption, freeze-drying was the least economical method in our study design, with a drying time of 27 h and an energy consumption of 4.69 EUR/kg mealworm larvae. In terms of energy costs, the other drying techniques followed the order microwave drying (2.16 EUR/kg mealworm larvae) > infrared drying (0.99 EUR/kg mealworm larvae) > rack oven drying (0.88 EUR/kg mealworm larvae) > high frequency drying (0.09 EUR/kg mealworm larvae). With the future goal of making the insect production value chain more sustainable, cost-efficient and less labour-intensive, it would be of great interest to evaluate the drying methods used here and other variants currently under development (e.g., low-energy electron beam drying in the SUSINCHAIN project) under large-scale production conditions with regard to economics, food safety and nutritional quality. The findings of this study on drying-induced changes in mealworm larvae will contribute to developing strategies to optimally process the insects from nutritive, safety and economic perspectives.

## Figures and Tables

**Table 1:** Energy demand of the different drying methods.

Drying Method	D_Temp_ (°C)	D_Temp_ (°C)	MO (kW)	t (h)	EC (kWh)	Larvae Material (kg)	EC/kg Larvae Material (kWh/kg) *	Energy Cost (EUR /kg Larvae Material) #
**Infrared drying**	130	130	22	0.25	5.5	1	5.5	0.99
**Rack oven drying**	60	250	0.43	19	1.96	0.4	4.9	0.88
	**P** **(kW)**			**t** **(h)**	**EC** **(kWh)**	**larvae material** **(kg)**	**EC/kg larvae** material (kWh/kg) *	**Energy cost** **(EUR /kg larvae material) #**
**Freeze-drying**	0.51			27	13.8	0.5	27.5	4.96
**Microwave drying**	7.2			0.25	1.8	0.15	12.0	2.16
**High frequency drying**	Real-time online measurement of energy consumption			1	0.27	0.54	0.5	0.09

* The calculation is based on the assumption that the equipment runs at maximal capacity during drying. **#** The calculation of the energy demand is based on the 4th report on energy prices and costs published by the European Commission in October 2020 [53]. The electricity price for non-households in the EU of EUR 0.18 per kWh was used.

**Table 2:** Nutritional values of fresh and dried mealworm larvae.

Drying Method	Undried	High-Frequency Dried	Rack Oven Dried	Infrared Dried	Microwave Dried	Freeze- Dried
**Water activity**	0.69 ± 0.00 ^a^	0.30 ± 0.00 ^b^	0.13 ± 0.01 ^c^	0.18 ± 0.01 ^d^	0.13 ± 0.01 ^c^	0.11 ± 0.00 ^e^
**Moisture** (g/100g)	59.53 ± 0.24 ^a^	1.38 ± 0.01 ^b^	7.87 ± 0.03 ^c^	2.23 ± 0.03 ^d^	2.67 ± 0.03 ^d^	5.47 ± 0.03 ^e^
**Protein** (g/100 g DM)	57.57 ± 1.09 ^a^	53.00 ± 1.80 ^b^	55.77 ± 0.07 ^a.b^	57.07 ± 0.03 ^a.b^	56.77 ± 0.07 ^a.b^	55.50 ± 0.15 ^a.b^
**Fat** (g/100 g DM)	26.80 ± 0.45 ^a^	23.07 ± 1.06 ^b^	28.83 ± 0.09 ^a^	27.47 ± 0.03 ^a^	27.47 ± 0.09 ^a^	28.43 ± 0.03 ^a^
**Ash** (g/100 g DM)	3.90 ± 0.00 ^a^	3.67 ± 0.09 ^a.b^	3.73 ± 0.07 ^a.c^	3.47 ± 0.03 ^b^	3.67 ± 0.03 ^a.b^	3.63 ± 0.03 ^b,c^

DM = mass of dried mealworms; Data are shown as means ± SEM of three replicates. Significantly different means within one row do not share the same letters (analysed by one-way ANOVA with Tukey’s multiple comparison test, *p* < 0.05).

**Table 3:** Colour parameters of dried mealworm larvae.

Colour Parameters	High-Frequency Dried	Rack Oven Dried	Infrared Dried	Microwave Dried	Freeze Dried
L*	33.00 ± 0.43 ^a^	41.27 ± 1.44 ^b^	50.27 ± 0.99 ^c^	60.60 ± 0.47 ^d^	66.13 ± 0.71 ^e^
a*	9.73 ± 0.05 ^a^	11.33 ± 0.11 ^a^	17.47 ± 0.52 ^b^	16.20 ± 0.16 ^b^	12.07 ± 0.20 ^c^
b*	19.00 ± 0.19 ^a^	20.13 ± 0.24 ^a^	36.00 ± 0.16 ^b^	46.93 ± 0.29 ^c^	44.47 ± 0.05 ^d^

Data are shown as means ± SEM of three replicates. Significantly different means within one row do not share the same letters (analysed by one-way ANOVA with Tukey’s multiple comparison test, *p* < 0.05).

**Table 4:** Fatty acid composition of dried mealworm larvae.

Fatty acid	High-Frequency Dried	Rack Oven Dried	Infrared Dried	Microwave Dried	Freeze- Dried
**Lauric acid** **(C12:0)**	0.31 ± 0.01 ^a^	0.34 ± 0.04 ^a^	0.30 ± 0.01 ^a^	0.26 ± 0.00 ^a^	0.20 ± 0.01 ^b^
**Myristic acid** **(C14:0)**	3.48 ± 0.14 ^a^	4.42 ± 0.01 ^b^	3.19 ± 0.02 ^a^	3.49 ± 0.02 ^a^	2.88 ± 0.05 ^c^
**Palmitic acid** **(C16:0)**	20.60 ± 0.14 ^a^	19.78 ± 0.15 ^b^	10.89 ± 0.09 ^c^	17.25 ± 0.02 ^d^	19.94 ± 0.16 ^b,e^
**Palmitoleic acid (C16:1 ω-7)**	1.34 ± 0.11 ^a^	1.46 ± 0.01 ^b^	1.77 ± 0.01 ^a^	1.57 ± 0.01 ^a^	1.47 ± 0.02 ^a^
**Stearic acid** **(C18:0)**	8.00 ± 0.38 ^a^	3.45 ± 0.01 ^b^	3.39 ± 0.01 ^b^	2.18 ± 0.06 ^c^	4.48 ± 0.01 ^d^
**Oleic acid** **(C18:1 ω-9)**	40.73 ± 0.13 ^a^	36.78 ± 0.21 ^b^	36.82 ± 0.12 ^b^	43.76 ± 0.03 ^c^	32.82 ± 0.04 ^d^
**Linoleic acid** **(C18:2 ω-6)**	25.54 ± 1.10 ^a^	31.91 ± 0.12 ^b^	41.27 ± 0.16 ^c^	30.18 ± 0.02 ^b^	35.89 ± 0.14 ^d^
**Linolenic acid** **(C18:3 ω-3)**	n.d.	1.12 ± 0.01 ^a^	1.18 ± 0.03 ^b^	1.01 ± 0.01 ^c^	1.48 ± 0.02 ^d^
**Arachidic acid** **(C20:0)**	n.d.	0.34 ± 0.01 ^a^	0.22 ± 0.01 ^b^	0.17 ± 0.01 ^c^	0.23 ± 0.02 ^b^
**Behenic acid** **(C22:0)**	n.d.	0.33 ± 0.01 ^a^	0.22 ± 0.01 ^a^	0.08 ± 0.01 ^b^	0.35 ± 0.04 ^c^
**Lignoceric acid** **(C24:0)**	n.d.	0.21 ± 0.01 ^a,b^	0.19 ± 0.01 ^a^	0.06 ± 0.01 ^c^	0.27 ± 0.02 ^b^
**∑SFA**	32.38 ± 0.27 ^a^	28.74 ± 0.10 ^b^	18.34 ± 0.07 ^c^	23.48 ± 0.07 ^d^	28.35 ± 0.17 ^b^
**∑MUFA**	42.07 ± 1.34 ^a^	38.24 ± 0.20 ^b^	38.59 ± 0.14 ^b^	45.33 ± 0.04 ^c^	34.29 ± 0.03 ^d^
**∑PUFA**	25.54 ± 1.10 ^a^	33.02 ± 0.13 ^b^	43.07 ± 0.17 ^c^	31.19 ± 0.04 ^b^	37.36 ± 0.15 ^d^
**∑UFA**	67.62 ± 0.28 ^a^	71.26 ± 0.09 ^b^	81.66 ± 0.07 ^c^	76.52 ± 0.04 ^d^	71.65 ± 0.17 ^b^
**PUFA/SFA**	0.78 ± 0.03^a^	1.15 ± 0.01^b^	2.35 ± 0.02^c^	1.33 ± 0.01^d^	1.2 ± 0.01^d^
**Peroxide value**	0.69 ± 0.05 ^a^	1.31 ± 0.07 ^a^	1.28 ± 0.22 ^a^	1.92 ± 0.35 ^a^	7.22 ± 0.78 ^b^

Fatty acid composition in percen total fatty acids; n.d. = not detected; SFA = saturated fatty acids; MUFA = monounsaturated fatty acids; PUFA = polyunsaturated fatty acids; UFA = unsaturated fatty acids. Peroxide values are expressed in mmol O_2_/kg fat. Data are shown as means ± SEM of three replicates. Significantly different means within one row do not share the same letters (analyzed by one-way ANOVA with Tukey’s multiple comparison test, *p* < 0.05).

## Data Availability

The data presented in this study are available on request from the corresponding author.

## Supplementary Materials

The following supporting information can be downloaded at: https://www.mdpi.com/article/10.3390/insects13020166/s1, Figure S1: Real-time monitoring of *T. molitor* larvae high-frequency drying, Table S1: Tabular representation of the static headspace GC-MS analysis of *T. molitor* larvae, Table S2: Tabular representation of the SPME headspace GC-MS analysis of *T. molitor* larvae, Table S3: Tabular representation of the ITEX headspace GC-MS analysis of *T. molitor* larvae.

## References

[1] United Nations (2019). Department of Economic and Social Affairs, Population Dynamics. World Population Prospects 2019.

[2] White R.R., Hall M.B. (2017). Nutritional and greenhouse gas impacts of removing animals from US agriculture. Proc. Natl. Acad. Sci. USA.

[3] Collins C.M., Vaskou P., Kountouris Y. (2019). Insect Food Products in the Western World: Assessing the Potential of a New “Green” Market. Ann. Entomol. Soc. Am..

[4] Alexandratos N., Bruinsma J. (2012). World Agriculture Towards 2030/2050: The 2012 Revision.

[5] Van Meerbeek K., Svenning J.C. (2018). Causing confusion in the debate about the transition toward amore plant-based diet. Proc. Natl. Acad. Sci. USA.

[6] Mazac R., Renwick K., Seed B., Black J.L. (2021). An Approach for Integrating and Analyzing Sustainability in Food-Based Dietary Guidelines. Front. Sustain. Food Syst..

[7] Rumpold B.A., Schlüter O.K. (2013). Nutritional composition and safety aspects of edible insects. Mol. Nutr. Food Res..

[8] Van Huis A., Rumpold B., Maya C., Roos N. (2021). Nutritional Qualities and Enhancement of Edible Insects. Annu. Rev. Nutr..

[9] Ojha S., Bekhit A.E.-D., Grune T., Schlüter O.K. (2021). Bioavailability of nutrients from edible insects. Curr. Opin. Food Sci..

[10] van Huis A., van Itterbeeck J., Klunder H., Mertens E., Halloran A., Muir G., Vantomme P. (2013). Future Prospects for Food and Feed Security.

[11] Wade M., Hoelle J. (2019). A review of edible insect industrialization: Scales of production and implications for sustainability. Environ. Res. Lett..

[12] Pippinato L., Gasco L., Di Vita G., Mancuso T. (2020). Current scenario in the European edible-insect industry: A preliminary study. J. Insects Food Feed.

[13] Lähteenmäki-Uutela A., Marimuthu S.B., Meijer N. (2021). Regulations on insects as food and feed: A global comparison. J. Insects Food Feed.

[14] Montanari F., Pinto de Moura A., Cunha L.M. (2021). Production and Commercialization of Insects as Food and Feed.

[15] EFSA Panel on Nutrition, N.F. and F.A. (NDA) (2021). Safety of dried yellow mealworm (Tenebrio molitor larva) as a novel food pursuant to Regulation (EU) 2015/2283. EFSA J..

[16] EFSA Panel on Nutrition, N.F. and F.A. (NDA) (2021). Safety of frozen and dried formulations from whole yellow mealworm (Tenebrio molitor larva) as a novel food pursuant to Regulation (EU) 2015/2283. EFSA J..

[17] EFSA (2015). Risk profile related to production and consumption of insects as food and feed. EFSA J..

[18] Cortes Ortiz J.A., Ruiz A.T., Morales-Ramos J.A., Thomas M., Rojas M.G., Tomberlin J.K., Yi L., Han R., Giroud L., Jullien R.L., Dossey A.T., Morales-Ramos J.A., Guadalupe Rojas M. (2016). Insect Mass Production Technologies. Insects as Sustainable Food Ingredients.

[19] Kröncke N., Baur A., Böschen V., Demtröder S., Benning R., Delgado A., Mariod A.A. (2020). Automation of Insect Mass Rearing and Processing Technologies of Mealworms (Tenebrio molitor). African Edible Insects as Alternative Source of Food, Oil, Protein and Bioactive Components.

[20] Nava A.L., Higareda T.E., Barreto C., Rodríguez R., Márquez I., Palacios M.L. (2020). Circular economy approach for mealworm industrial production for human consumption. IOP Conf. Ser. Earth Environ. Sci..

[21] Rumpold B.A., Schlüter O.K. (2013). Potential and challenges of insects as an innovative source for food and feed production. Innov Food Sci Emerg Technol..

[22] Schrögel P., Wätjen W. (2019). Insects for food and feed-safety aspects related to mycotoxins and metals. Foods.

[23] Son Y.J., Hwang I.K., Nho C.W., Kim S.M., Kim S.H. (2021). Determination of carbohydrate composition in mealworm (Tenebrio molitor l.) larvae and characterization of mealworm chitin and chitosan. Foods.

[24] Kröncke N., Böschen V., Woyzichovski J., Demtröder S., Benning R. (2018). Comparison of suitable drying processes for mealworms (Tenebrio molitor). Innov. Food Sci. Emerg. Technol..

[25] Van Boekel M.A.J.S. (2006). Formation of flavour compounds in the Maillard reaction. Biotechnol. Adv..

[26] Kanzler C., Haase P.T., Schestkowa H., Kroh L.W. (2016). Antioxidant Properties of Heterocyclic Intermediates of the Maillard Reaction and Structurally Related Compounds. J. Agric. Food Chem..

[27] Lund M.N., Ray C.A. (2017). Control of Maillard Reactions in Foods: Strategies and Chemical Mechanisms. J. Agric. Food Chem..

[28] Frankel E.N. (2005). Lipid Oxidation: Second Edition.

[29] Grebenteuch S., Kroh L.W., Drusch S., Rohn S. (2021). Formation of secondary and tertiary volatile compounds resulting from the lipid oxidation of rapeseed oil. Foods.

[30] Zamora R., Hidalgo F.J. (2007). Coordinate Contribution of Lipid Oxidation and Maillard Reaction to the Nonenzymatic Food Browning. Crit. Rev. Food Sci. Nutr..

[31] Ojha S., Bußler S., Psarianos M., Rossi G., Schlüter O.K. (2021). Edible insect processing pathways and implementation of emerging technologies. J. Insects Food Feed.

[32] Melgar-Lalanne G., Hernández-Álvarez A.J., Salinas-Castro A. (2019). Edible Insects Processing: Traditional and Innovative Technologies. Compr. Rev. Food Sci. Food Saf..

[33] International Platform of Insects for Food and Feed (IPIFF) IPIFF Guide on Good Hygiene Practices for European Union (EU) Producers of Insects as Food and Feed-Updated October 2021. https://ipiff.org/good-hygiene-practices/.

[34] (2020). SUSINCHAIN SUStainable INsect CHAIN (SUSINCHAIN) - Newsletter. https://susinchain.eu/newsletter/1/.

[35] Selaledi L., Mabelebele M. (2021). The influence of drying methods on the chemical composition and body color of yellow mealworm (Tenebrio molitor L.). Insects.

[36] Seho R.E.Y., Monteiro R.L., De Dea Lindner J., Miotto M., Carciofi B.A.M., Laurindo J.B. (2022). Effects of vacuum and multiflash drying on the microbiota and colour of dried yellow mealworm ( Tenebrio molitor ). J. Insects Food Feed.

[37] Kröncke N., Grebenteuch S., Keil C., Demtröder S., Kroh L., Thünemann A., Benning R., Haase H., Kröncke N., Grebenteuch S. (2019). Effect of Different Drying Methods on Nutrient Quality of the Yellow Mealworm (Tenebrio molitor L.). Insects.

[38] Lenaerts S., Van Der Borght M., Callens A., Van Campenhout L. (2018). Suitability of microwave drying for mealworms (Tenebrio molitor) as alternative to freeze drying: Impact on nutritional quality and colour. Food Chem..

[39] Purschke B., Brüggen H., Scheibelberger R., Jäger H. (2018). Effect of pre-treatment and drying method on physico-chemical properties and dry fractionation behaviour of mealworm larvae (Tenebrio molitor L.). Eur. Food Res. Technol..

[40] Hernández-Álvarez A.J., Mondor M., Piña-Domínguez I.A., Sánchez-Velázquez O.A., Melgar Lalanne G. (2021). Drying technologies for edible insects and their derived ingredients. Dry. Technol..

[41] Sindermann D., Heidhues J., Kirchner S., Stadermann N., Kuhl A. (2021). Industrial processing technologies for insect larvae. J. Insects Food Feed.

[42] Saifullah M., McCullum R., McCluskey A., Vuong Q. (2019). Effects of different drying methods on extractable phenolic compounds and antioxidant properties from lemon myrtle dried leaves. Heliyon.

[43] Verband Deutscher Landwirtschaftlicher Untersuchungs-und Forschungsanstalten VDLUFA methodenbuch III (2013). VDLUFA-Verlag (Vol. Ed.), Band III-Die Chemische Untersuchung von Futtermitteln.

[44] de Oliveira L.M., da Silva Lucas A.J., Oliveira F.G. (2018). Evaluation of Color Tenebrio molitor Larvae by Different Methods of Dehydration. J. Food Process. Technol..

[45] Folch J., Lees M., Sloane Stanley G.H. (1957). A simple method for the isolation and purification of total lipides from animal tissues. J. Biol. Chem..

[46] Fiebig H.-J., Godelmann R. (1997). Bestimmung der Peroxidzahl (Methode nach Wheeler) - Deutsche Einheitsmethoden zur Untersuchung von Fetten, Fettprodukten, Tensiden und verwandten Stoffen: Analyse von Fetten XXXVII. Lipid / Fett.

[47] Kremser A., Jochmann M.A., Schmidt T.C. (2016). Systematic comparison of static and dynamic headspace sampling techniques for gas chromatography. Anal. Bioanal. Chem..

[48] Ilyasov I.R., Beloborodov V.L., Selivanova I.A., Terekhov R.P. (2020). ABTS/PP decolorization assay of antioxidant capacity reaction pathways. Int. J. Mol. Sci..

[49] Kanzler C., Schestkowa H., Haase P.T., Kroh L.W. (2017). Formation of Reactive Intermediates, Color, and Antioxidant Activity in the Maillard Reaction of Maltose in Comparison to d -Glucose. J. Agric. Food Chem..

[50] Apak R., Özyürek M., Güçlü K., Esra Çapanoğlu Antioxidant activity/capacity measurement (2016). 1. Classification, physicochemical principles, mechanisms, and electron transfer (ET)-based assays. J. Agric. Food Chem..

[51] Kedare S.B., Singh R.P. (2011). Genesis and development of DPPH method of antioxidant assay. J. Food Sci. Technol..

[52] Ainsworth E.A., Gillespie K.M. (2007). Estimation of total phenolic content and other oxidation substrates in plant tissues using Folin-Ciocalteu reagent. Nat. Protoc..

[53] (2020). EU Report From The Commission to the European Parliament, the Council, the European Economic and Social Committee and the Committee of the Regions Energy prices and costs in Europe; COM/2020/951 final. https://eur-lex.europa.eu/legal-content/EN/TXT/PDF/?uri=CELEX:52016DC0805&from=EN.

[54] (2017). Jongema Y List of edible insects of the world. https://www.wur.nl/en/Research-Results/Chair-groups/Plant-Sciences/Laboratory-of-Entomology/Edible-insects/Worldwide-species-list.htm.

[55] Gkinali A.-A., Matsakidou A., Vasileiou E., Paraskevopoulou A. (2022). Potentiality of Tenebrio molitor larva-based ingredients for the food industry: A review. Trends Food Sci. Technol..

[56] Errico S., Spagnoletta A., Verardi A., Moliterni S., Dimatteo S., Sangiorgio P. (2021). Tenebrio molitor as a source of interesting natural compounds, their recovery processes, biological effects, and safety aspects. Compr. Rev. Food Sci. Food Saf..

[57] Rezaei F., vander Gheynst J.S. (2010). Critical moisture content for microbial growth in dried food-processing residues. J. Sci. Food Agric..

[58] Azzollini D., Derossi A., Severini C. (2016). Understanding the drying kinetic and hygroscopic behaviour of larvae of yellow mealworm (Tenebrio molitor) and the effects on their quality. J. Insects as Food Feed..

[59] Jiao Y., Tang J., Wang Y., Koral T.L. (2018). Radio-Frequency Applications for Food Processing and Safety. Annu. Rev. Food Sci. Technol..

[60] (2021). SUSINCHAIN Reporting Periodic Reporting for Period 1-SUSINCHAIN (SUStainable INsect CHAIN). https://cordis.europa.eu/project/id/861976/reporting.

[61] Chaowattanakul T., Khieu V.M., Rojviriya C., Siriwong S., Jittanit W., Chanput W.P. (2021). Energy consumption, physical properties, protein structure and digestibility of edible insects dried using three methods. J. Insects Food Feed.

[62] Lawal K.G., Kavle R.R., Akanbi T.O., Mirosa M., Agyei D. (2021). Enrichment in specific fatty acids profile of Tenebrio molitor and Hermetia illucens larvae through feeding. Future Foods.

[63] (2010). EFSA Scientific Opinion on Dietary Reference Values for fats, including saturated fatty acids, polyunsaturated fatty acids, monounsaturated fatty acids, trans fatty acids, and cholesterol. EFSA J..

[64] Grebenteuch S., Kanzler C., Klaußnitzer S., Kroh L.W., Rohn S. (2021). The formation of methyl ketones during lipid oxidation at elevated temperatures. Molecules.

[65] Ramos P.R., Characterisation of Volatile Compounds Found in Tenebrio Molitor l (2020). Reared with Different Dietary Regimes Using Headspace Solid-Phase Microextraction. Northumbria University Newcastle.

[66] Halarnkar P.P., Schooley D.A. (1995). A comparative catabolism study of isoleucine by insect and mammalian tissues. Comp. Biochem. Physiol. Part B Biochem..

[67] Smit B.A., Engels W.J.M., Smit G. (2009). Branched chain aldehydes: Production and breakdown pathways and relevance for flavour in foods. Appl. Microbiol. Biotechnol..

[68] Hidalgo F.J., León M.M., Zamora R. (2016). Amino acid decarboxylations produced by lipid-derived reactive carbonyls in amino acid mixtures. Food Chem..

[69] Hofmann T., Münch P., Schieberle P. (2000). Quantitative model studies on the formation of aroma-active aldehydes and acids by Strecker-type reactions. J Agric Food Chem..

[70] Flament I. (2002). Coffee Flavor Chemistry.

[71] Grossmann K.K., Merz M., Appel D., De Araujo M.M., Fischer L. (2021). New insights into the flavoring potential of cricket (Acheta domesticus) and mealworm (Tenebrio molitor) protein hydrolysates and their Maillard products. Food Chem..

[72] Elhassan M., Wendin K., Olsson V., Langton M., Elhassan M., Wendin K., Olsson V., Langton M. (2019). Quality Aspects of Insects as Food—Nutritional, Sensory, and Related Concepts. Foods.

[73] Seo H., Kim H.R., Cho I.H. (2020). Aroma characteristics of raw and cooked tenebrio molitor larvae (mealworms). Food Sci. Anim. Resour..

[74] Żołnierczyk A.K., Szumny A. (2021). Sensory and chemical characteristic of two insect species: Tenebrio molitor and zophobas morio larvae affected by roasting processes. Molecules.

[75] D’Antonio V., Serafini M., Battista N. (2021). Dietary Modulation of Oxidative Stress From Edible Insects: A Mini-Review. Front. Nutr..

[76] Mancini S., Fratini F., Turchi B., Mattioli S., Dal Bosco A., Tuccinardi T., Nozic S., Paci G. (2019). Former foodstuff products in Tenebrio molitor rearing: Effects on growth, chemical composition, microbiological load, and antioxidant status. Animals.

[77] Baek M., Kim M.A., Kwon Y.S., Hwang J.S., Goo T.W., Jun M., Yun E.Y. (2019). Effects of processing methods on nutritional composition and antioxidant activity of mealworm (Tenebrio molitor) larvae. Entomol. Res..

[78] Mancini S., Mattioli S., Paolucci S., Fratini F., Dal Bosco A., Tuccinardi T., Paci G. (2021). Effect of Cooking Techniques on the in vitro Protein Digestibility, Fatty Acid Profile, and Oxidative Status of Mealworms (Tenebrio molitor). Front. Vet. Sci..

[79] Di Mattia C., Battista N., Sacchetti G., Serafini M. (2019). Antioxidant activities in vitro of water and liposoluble extracts obtained by different species of edible insects and invertebrates. Front. Nutr..

[80] Navarro del Hierro J., Gutiérrez-Docio A., Otero P., Reglero G., Martin D. (2020). Characterization, antioxidant activity, and inhibitory effect on pancreatic lipase of extracts from the edible insects Acheta domesticus and Tenebrio molitor. Food Chem..

[81] Finke M.D. (2015). Complete nutrient content of four species of commercially available feeder insects fed enhanced diets during growth. Zoo Biol..

[82] Everette J.D., Bryant Q.M., Green A.M., Abbey Y.A., Wangila G.W., Walker R.B. (2010). Thorough study of reactivity of various compound classes toward the folin-Ciocalteu reagent. J. Agric. Food Chem..

[83] Jeong M.K., Yeo J.D., Jang E.Y., Kim M.J., Lee J.H. (2012). Aldehydes from oxidized lipids can react with 2,2-diphenyl-1-picrylhydrazyl (DPPH) free radicals in isooctane systems. JAOCS J. Am. Oil Chem. Soc..

[84] Cämmerer B., Chodakowski K., Gienapp C., Wohak L., Hartwig A., Kroh L.W. (2012). Pro-oxidative effects of melanoidin-copper complexes on isolated and cellular DNA. Eur. Food Res. Technol..

[85] Fiol M., Weckmüller A., Neugart S., Schreiner M., Rohn S., Krumbein A., Kroh L.W. (2013). Thermal-induced changes of kale’s antioxidant activity analyzed by HPLC-UV/Vis-online-TEAC detection. Food Chem..

[86] Mocan A., Schafberg M., Crisan G., Rohn S. (2016). Determination of lignans and phenolic components of Schisandra chinensis (Turcz.) Baill. using HPLC-ESI-ToF-MS and HPLC-online TEAC: Contribution of individual components to overall antioxidant activity and comparison with traditional antioxidant assays. J. Funct. Foods.

