# Supplementary material for: Systematic Studies on the Antioxidant Capacity and Volatile Compound Profile of Yellow Mealworm Larvae (T. molitor L.) under Different Drying Regimes
Source: Insects. 2022 Feb 3;13(2):166. doi: 10.3390/insects13020166 (PMC8876196; doi:10.3390/insects13020166)
Supplement: Supplementary file 1 [file insects-13-00166-s001.zip › insects-1575982-supplementary.pdf]

Systematic Studies on the Antioxidant Capacity and Volatile Compound Profile of Yellow Mealworm Larvae (*T. molitor* L.) under Different Drying Regimes

Claudia Keil [1,*,†], Sandra Grebenteuch [2,3,†], Nina Kröncke [4], Fenja Kulow [1], Sebastian Pfeif [2], Clemens Kanzler [2], Sascha Rohn [2,3], Georg Boeck [5], Rainer Benning [4] and Hajo Haase [1,*]

1 Department of Food Chemistry and Toxicology, Institute of Food Technology and Food Chemistry,
Technische Universität Berlin, Straße des 17. Juni 135, 10623 Berlin, Germany; fenja.kulow@yahoo.de

2 Department of Food Chemistry and Analysis, Institute of Food Technology and Food Chemistry,
Technische Universität Berlin, Straße des 17. Juni 135, 10623 Berlin, Germany; sandra.grebenteuch@tu-berlin.de (S.G.); pfeifsebastian@gmail.com (S.P.); clemens.kanzler@tu-berlin.de (C.K.); rohn@tu-berlin.de (S.R.)

3 Institute for Food and Environmental Research e. V., Papendorfer Weg 3, 14806 Bad Belzig, Germany

4 Institute of Food Technology and Bioprocess Engineering, University of Applied Sciences Bremerhaven, An der Karlstadt 8, 27568 Bremerhaven, Germany; nkroencke@hs-bremerhaven.de (N.K.); rbenning@hs-bremerhaven.de (R.B.)

5 GloMic GmbH, Krampnitzer Weg 102, 14089 Berlin, Germany; g.boeck@glomic.de

* Correspondence: c.keil@tu-berlin.de (C.K.); haase@tu-berlin.de (H.H.); Tel.: +49-(0)-30-31472816 (C.K.);
+49-(0)-30-31472701 (H.H.); Fax: +49-(0)-30-31472823 (C.K. & H.H.)

† These authors contributed equally to this work.

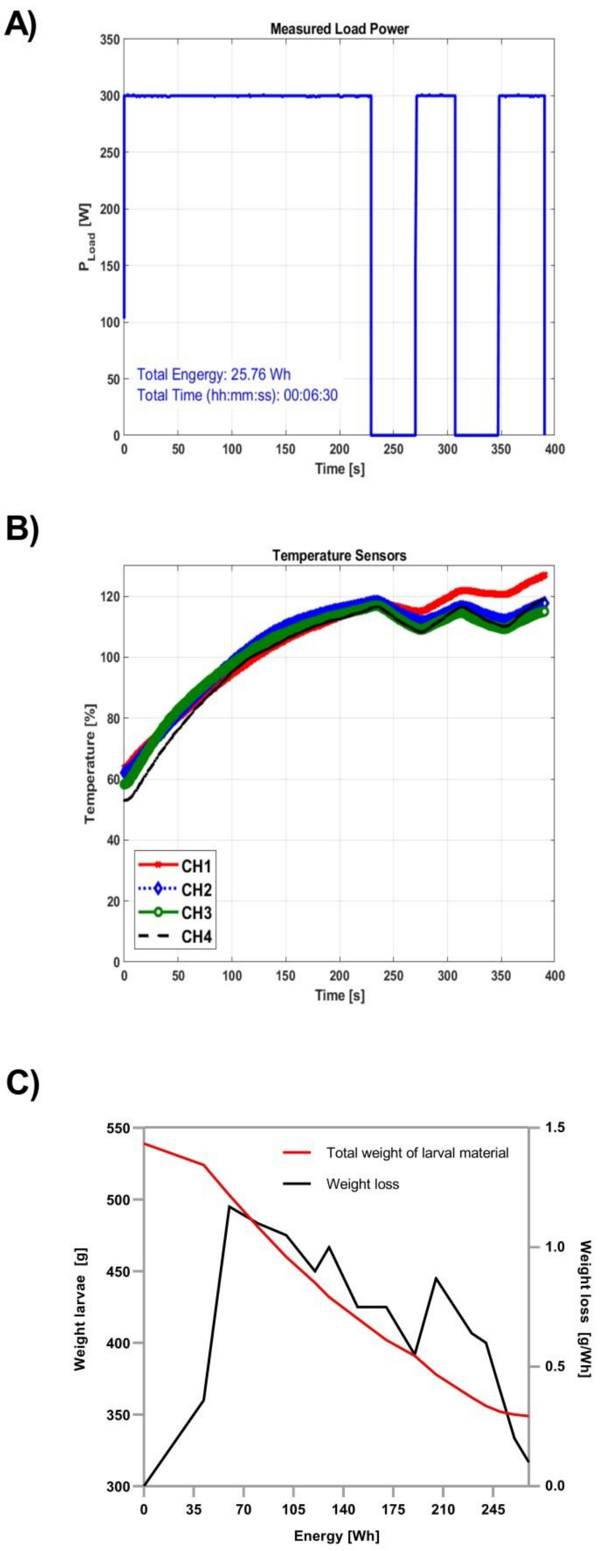

Suppl. Figure S1. Real-time monitoring of *T. molitor* larvae high-frequency drying.

Mealworm samples were subjected to a 1-hour high-frequency treatment with integrated cooling pauses to avoid overheating. Power load (A) and temperature profile (B) during the initial heating phase and first heating/cooling cycles. (C) Weight loss of the larvae material during the 1-hour high-frequency drying process.

Suppl. Table S1. Tabular representation of the static headspace GC-MS analysis of *T. molitor* larvae.

	Chemical Group	Volatile compound	Peak area [AU × 10^5]					
			**No dry**	**High-frequency dried**	**Rack oven dried**	**Infrared dried**	**Microwave dried**	**Freeze dried**
**1**	Aldehyde	Pentanal	n.d.	2.00 ± 0.23	n.d.	2.12 ± 0.24	2.08 ± 0.29	33.43 ± 1.62
**2**	Aldehyde	Hexanal	5.74 ± 0.23	7.58 ± 0.21	3.39 ± 0.26	9.51 ± 0.46	6.70 ± 1.12	16.25 ± 10.87
**3**	Aldehyde	Heptanal	n.d.	0.76 ± 0.05	n.d.	0.88 ± 0.07	0.51 ± 0.09	4.52 ± 0.22
**4**	Aldehyde	Octanal	n.d.	0.57 ± 0.06	n.d.	0.27 ± 0.11	0.36 ± 0.10	2.68 ± 0.07
**5**	Aldehyde	Nonanal	0.94 ± 0.01	1.33 ± 0.11	n.d.	1.05 ± 0.01	0.45 ± 0.17	2.72 ± 0.10
**6**	Alkane	n-Octan	n.d.	n.d.	n.d.	n.d.	0.54 ± 0.33	10.09 ± 0.64
**7**	Ketone	2-Butanon	n.d.	4.77 ± 0.44	n.d.	n.d.	4.77 ± 0.44	3.10 ± 0.73
**8**	Ketone	2-Heptanone	n.d.	0.37 ± 0.04	n.d.	1.27 ± 0.12	0.84 ± 0.17	9.82 ± 0.39
**9**	Furan	2-Butylfuran	n.d.	n.d.	n.d.	n.d.	n.d.	0.93 ± 0.09
**10**	Furan	2-Pentylfuran	0.64 ± 0.04	0.54 ± 0.01	n.d.	0.46 ± 0.00	0.28 ± 0.25	13.10 ± 1.48
**11**	Aldehyde	2-Butyl-2-octenal	n.d.	n.d.	n.d.	n.d.	n.d.	5.90 ± 0.1
**12**	Acid	Acetic acid	n.d.	n.d.	50.43 ± 1.40	31.59 ± 15.24	7.55 ± 4.56	n.d.
**13**	Aldehyde	2-Methylpropanal	128.17 ± 15.41	181.62 ± 14.02	165.33 ± 13.82	133.64 ± 14.26	73.97 ± 6.92	429.77 ± 20.94
**14**	Aldehyde	3-Methylbutanal	245.50 ± 32.59	203.08 ± 14.06	255.42 ± 14.31	79.84 ± 5.56	31.10 ± 5.19	252.31 ± 9.59
**15**	Aldehyde	2-Methylbutanal	112.59 ± 16.57	142.78 ± 8.67	153.30 ± 8.36	89.26 ± 4.27	49.13 ± 6.82	277.91 ± 11.56
**16**	Aldehyde	Benzenacetaldehyde	2.19 ± 0.12	0.86 ± 0.02	1.40 ± 0.11	n.d.	n.d.	n.d.
**17**	Acid	2-Methylpropanoic acid	n.d.	n.d.	4.01 ± 1.05	n.d.	n.d.	n.d.
**18**	Acid	3-Methylbutanoic acid	n.d.	n.d.	10.40 ± 2.01	n.d.	n.d.	n.d.
**19**	Acid	2-Methylbutanoic acid	n.d.	n.d.	2.69 ± 0.51	n.d.	n.d.	n.d.
**20**	Pyrazine	2-Methylpyrazine	n.d.	0.25 ± 0.01	0.51 ± 0.01	n.d.	0.28 ± 0.01	n.d.
**21**	Pyrazine	2,5-Dimethylpyrazine	n.d.	0.38 ± 0.16	0.28 ± 0.10	0.31 ± 0.01	0.74 ± 0.28	n.d.
**22**	Chloroalkane	Chloroform	n.d.	3.12 ± 0.29	n.d.	32.92 ± 1.48	120.07 ± 12.52	55.10 ± 4.00
**23**	Aldehyde	Benzaldehyde	0.92 ± 0.14	1.34 ± 0.01	0.88 ± 0.11	0.70 ± 0.01	0.57 ± 0.01	1.00 ± 0.10
**24**	Terpene	Limonen	3.79 ± 0.01	2.17 ± 0.10	1.48 ± 0.01	2.33 ± 0.15	2.98 ± 0.41	4.70 ± 0.01

Mean ± SEM (N ≥3) of the peak area of volatile organic compounds relative to larval weight; white: Lipid oxidation intermediates; gray: Maillard intermediates, black: others; n.d. = not detected

Suppl. Table S2. Tabular representation of the SPME headspace GC-MS analysis of *T. molitor* larvae.

			Peak area [AU × 10^5]					
	**Chemical Group**	**Volatile compound**	**No dry**	**High-frequency dried**	**Rack oven dried**	**Infrared dried**	**Microwave dried**	**Freeze dried**
**1**	Aldehyde	Pentanal	n.d.	n.d.	n.d.	n.d.	n.d.	1.42 ± 0.37
**2**	Aldehyde	Hexanal	1.64 ± 0.13	1.06 ± 0.01	n.d.	0.20 ± 0.17	0.93 ± 0.14	3024298 ± 442717
**3**	Aldehyde	Heptanal	n.d.	n.d.	n.d.	n.d.	n.d.	1.18 ± 0.28
**4**	Aldehyde	Octanal	n.d.	n.d.	n.d.	n.d.	n.d.	1.41 ± 0.24
**5**	Aldehyde	Nonanal	2.97 ± 0.25	n.d.	0.70 ± 0.01	0.78 ± 0.13	n.d.	0.20 ± 0.34
**25**	Acid	Hexanoic acid	n.d.	n.d.	n.d.	n.d.	n.d.	7.79 ± 3.31
**6**	Alkane	n-Octan	n.d.	n.d.	n.d.	n.d.	n.d.	1.72 ± 0.40
**8**	Ketone	2-Heptanone	n.d.	n.d.	n.d.	0.78 ± 0.01	0.44 ± 0.01	3.64 ± 0.21
**9**	Furan	2-Butylfuran	n.d.	n.d.	n.d.	n.d.	n.d.	0.26 ± 0.01
**10**	Furan	2-Pentylfuran	0.83 ± 0.15	0.40 ± 0.00	n.d.	0.43 ± 0.00	n.d..	9.23 ± 1.12
**11**	Aldehyde	2-Butyl-2-octenal	n.d.	n.d.	n.d.	n.d.	n.d.	24.95 ± 1.57
**12**	Acid	Acetic acid	n.d.	25.36 ± 1.48	1.67 ± 0.49	12.59 ± 1.11	n.d.	8.06 ± 1.36
**13**	Aldehyde	2-Methylpropanal	1.66 ± 0.54	2.69 ± 0.50	n.d.	n.d.	2.48 ± 0.16	n.d.
**14**	Aldehyde	3-Methylbutanal	20.65 ± 0.76	8.89 ± 0.42	1.46 ± 0.20	5.42 ± 0.27	2.02 ± 0.06	10.88 ± 0.33
**15**	Aldehyde	2-Methylbutanal	7.90 ± 0.59	5.94 ± 0.47	1.19 ± 0.37	8.53 ± 0.40	3.12 ± 0.19	15.06 ± 0.40
**16**	Aldehyde	Benzenacetaldehyde	5.24 ± 0.52	n.d.	1.42 ± 0.01	n.d.	n.d.	n.d.
**17**	Acid	2-Methylpropanoic acid	n.d.	n.d.	6.57 ± 0.35	n.d.	n.d.	n.d.
**18**	Acid	3-Methylbutanoic acid	n.d.	n.d.	34.90 ± 0.32	n.d.	n.d.	n.d.
**19**	Acid	2-Methylbutanoic acid	n.d.	n.d.	11.85 ± 0.28	n.d.	n.d.	n.d.
**20**	Pyrazine	2-Methylpyrazine	n.d.	1.33 ± 0.01	1.42 ± 0.01	1.04 ± 0.01	0.36 ± 0.01	n.d.
**21**	Pyrazine	2,5-Dimethylpyrazine	n.d.	n.d.	n.d.	n.d.	n.d.	259315 ± 15289
**26**	Pyrazine	2,3,5-Trimethylpyrazine	n.d.	1.64 ± 0.14	n.d.	n.d.	0.50 ± 0.01	n.d.
**27**	Pyrazine	2-Ethyl-5-methylpyrazine	n.d.	123531 ± 8619	n.d.	n.d.	n.d.	n.d.
**28**	Pyrazine	2-Ethyl-3,6-dimethylpyrazine	n.d.	1.19 ± 0.15	0.72± 0.11	0.92 ± 0.01	0.50 ± 0.00	0.49 ± 0.01
**23**	Aldehyde	Benzaldehyde	1.34 ± 0.17	1.96 ± 0.01	2.07 ± 0.01	1.28 ± 0.12	0.53 ± 0.01	1.26 ± 0.29
**24**	Terpene	Limonen	5.25 ± 0.20	2.19 ± 0.01	0.25 ± 0.01	3.57 ± 0.14	5.94 ± 0.01	4.29 ± 0.21

Mean ± SEM (N ≥3) of the peak area of volatile organic compounds relative to larval weight; white: Lipid oxidation intermediates; gray: Maillard intermediates, black: others; n.d. = not detected.

Suppl. Table S3. Tabular representation of the ITEX headspace GC-MS analysis of *T. molitor* larvae.

			Peak area [AU × 10^5]					
	**Chemical Group**	**Volatile compound**	**No dry**	**High-frequency dried**	**Rack oven dried**	**Infrared dried**	**Microwave dried**	**Freeze dried**
**1**	Aldehyde	Pentanal	n.d.	7.84 ± 0.29	4.75 ± 0.34	7.14 ± 0.70	33.16 ± 3.49	175.69 ± 23.05
**2**	Aldehyde	Hexanal	14.46 ± 1.84	35.17 ± 1.03	16.65 ± 1.75	42.47 ± 2.26	4.72 ± 0.72	1112.08 ± 163.34
**3**	Aldehyde	Heptanal	1.30 ± 0.17	4.47 ± 0.23	2.30 ± 0.32	3.84 ± 0.28	1.05 ± 0.22	22.39 ± 2.55
**4**	Aldehyde	Octanal	0.67 ± 0.21	2.85 ± 0.17	1.23 ± 0.21	1.92 ± 0.19	27.69 ± 3.50	12.63 ± 1.93
**5**	Aldehyde	Nonanal	2.74 ± 0.51	6.28 ± 0.41	2.78 ± 0.27	3.10 ± 0.26	2.13 ± 0.41	6.68 ± 0.79
**29**	Aldehyde	2-Hexenal	n.d.	n.d.	n.d.	n.d.	n.d.	14.75 ± 1.41
**30**	Alcohol	1-Pentanol	n.d.	n.d.	n.d.	n.d.	n.d.	1.21 ± 2.32
**6**	Alkane	n-Octan	n.d.	n.d.	n.d.	6.55 ± 0.41	2.32 ± 0.41	74.92 ± 6.54
**7**	Ketone	2-Butanone	n.d.	9.84 ± 3.73	1.45 ± 1.29	6.18 ± 1.45	154.85 ± 14.47	39.39 ± 7.44
**8**	Ketone	2-Heptanone	0.13 ± 0.13	1.60 ± 0.01	1.60 ± 0.13	4.63 ± 0.35	6.13 ± 1.09	47.43 ± 4.42
**9**	Furan	2-Butylfuran	n.d.	n.d.	n.d.	n.d.	n.d.	7.51 ± 1.15
**10**	Furan	2-Pentylfuran	1.26 ± 0.01	2.38 ± 0.13	1.88 ± 0.27	1.85 ± 0.14	2.47 ± 0.40	71.34 ± 9.14
**11**	Aldehyde	2-Butyl-2-octenal	n.d.	n.d.	n.d.	n.d.	n.d.	1640446 ± 202490
**12**	Acid	Acetic acid	n.d.	18.82 ± 16.83	24.27 ± 20.12	96.62 ± 83.66	21.86 ± 2.45	n.d.
**13**	Aldehyde	2-Methylpropanal	139.23 ± 18.80	576.63 ± 45.95	474.64 ± 26.19	446.73 ± 20.15	310.60 ± 43.22	721.48 ± 88.65
**14**	Aldehyde	3-Methylbutanal	453.66 ± 67.53	848.72 ± 52.90	1027.06 ± 80.29	351.94 ± 8.41	243.61 ± 30.31	661.60 ± 33.35
**15**	Aldehyde	2-Methylbutanal	187.73 ± 31.72	529.71 ± 32.80	600.29 ± 52.61	374.00 ± 9.39	7.24 ± 2.64	698.91 ± 40.31
**16**	Aldehyde	Benzenacetaldehyde	27.15 ± 5.04	4.18 ± 0.23	8.19 ± 1.35	1.90 ± 0.27	n.d.	n.d.
**18**	Acid	3-Methylbutanoic acid	n.d.	n.d.	8.71 ± 5.96	0.56 ± 0.50	n.d.	n.d.
**19**	Acid	2-Methylbutanoic acid	n.d.	n.d.	2.96 ± 1.97	0.71 ± 0.63	n.d.	n.d.
**20**	Pyrazine	2-Methylpyrazine	n.d.	129278 ± 15031	443662 ± 64207	336729 ± 49429	288400 ± 31932	295427 ± 48574
**21**	Pyrazine	2,5-Dimethylpyrazine	n.d.	197103 ± 49013	826976 ± 144428	782072 ± 116276	148317 ± 23124	1026029 ± 179065
**28**	Pyrazine	2-Ethyl-3,6-dimethylpyrazine	n.d.	n.d.	n.d.	0.20 ± 0.17	n.d.	n.d.
**29**	Chloroalkane	Chloroform	n.d.	n.d.	57.99 ± 2.98	41.38 ± 3.06	155.57 ± 23.13	244.06 ± 9.04
**23**	Aldehyde	Benzaldehyde	3.35 ± 0.77	4.12 ± 0.22	4.29 ± 0.71	3.30 ± 0.27	0.27 ± 0.25	2.67 ± 0.31
**24**	Terpene	Limonen	8.58 ± 1.04	16.18 ± 0.40	24.31 ± 2.73	23.27 ± 1.59	0.88 ± 0.21	30.91 ± 3.06

Mean ± SEM (N ≥3) of the peak area of volatile organic compounds relative to larval weight; white: Lipid oxidation intermediates; gray: Maillard intermediates, black: others. n.d. = not detected.
